# Optimization of a plasmonic lens structure for maximum optical vortices induced on Weyl semimetal surface states†

**DOI:** 10.1039/d4na00336e

**Published:** 2024-09-10

**Authors:** Ritwik Banerjee, Tanmoy Maiti

**Affiliations:** a Plasmonics and Perovskites Laboratory, Indian Institute of Technology Kanpur UP 208016 India tmaiti@iitk.ac.in +91-512-259-6599

## Abstract

Optical vortices have a topologically charged phase singularity and zero intensity distribution in the centre. Optical vortex creation is regarded as a significant means for information transmission for applications in quantum computing, encryption, optical communication, *etc.* In this study, using finite-difference time-domain (FDTD) simulation, we calculated the electric field intensity and phase distribution of 2D lattices of optical vortices generated from various polygonal plasmonic lens structures using surface states of a Weyl semimetal (MoTe_2_). It was shown that a hexagonal lens is the best performing plasmonic lens. Further, we posited here a unified mathematical formulation for optical electrical field and phase distribution in the near field for any polygonal plasmonic lens. Our theoretical calculation corroborated well with FDTD results, validating the proposed generalized formula. Such plasmonic lens structures demonstrating scaling behavior offer great potential for designing next-generation optical memories.

## Abstract 2

A unified mathematical equation of a polygonal plasmonic lens is proposed, which can calculate the electric field intensity and phase-distribution maps of any polygonal lens.

## Graphical abstract

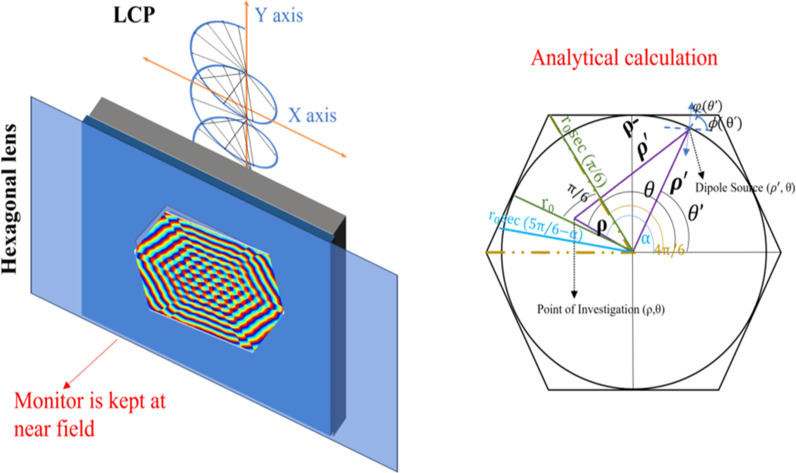

## Introduction

A vortex is a macroscopic phenomenon that can be seen in turbulent flow, tropical cyclones, smoke rings, liquid helium, and various other areas of hydrodynamics. Collet *et al.* reduced this macroscopic phenomenon to a microscopic concept by solving Maxwell–Bloch equations and introducing the concept of an optical vortex in 1989.^1^ Phase singularity in an optical vortex looks like an isolated dark patch with a unique phase in the centre holding some topological charges, dependent on how much light is twisted in a single wavelength.^2,3^ Momentum carried by an electro-magnetic wave like light comprises two components: spin angular momentum (SAM) and orbital angular momentum (OAM). Allen *et al.*^4^ proposed that OAM in vortex beams, where optical vortices propagate in paraxial beams, can be much greater than SAM. Since the development of optical vortices, their features, including detecting topological charges,^5^ optical communication in free space^6–11^ using different OAM modes, higher dimensional quantum entanglement,^12–14^ and microparticle manipulation,^15,16^ have stimulated the curiosity of researchers in a wide range of domains. Wang *et al.*'s work^7^ in particular piqued the scientific community's curiosity as soon as the study was published in 2012, with a national splash in a popular news e-portal headlined “Twisted light carries 2.5 terabits of data per second”. The researchers applied the notion of OAM multiplexing and demultiplexing, which is equivalent to wavelength-division multiplexing in fibre communication,^17–20^ to possibly transport up to 1369.6 Gbit s^−1^. This technique has the potential to have a tremendous influence on optical data communication,^9^ as data can be stored in massive amounts due to the unlimited range of possible OAM states. Khonina *et al.* further proposed a method for calculating information transmission^21^ in a group of optical vortices using spiral diffractive optical elements. The huge application potential of optical vortices has prompted scientists to fabricate optical lenses^22^ by combining two cylindrical lenses, thus enabling the conversion of the Hermite–Gaussian (HG) mode into the Laguerre–Gaussian (LG) mode and *vice versa*. Later, a group of scientists^23^ created a subwavelength periodic groove structure with a variable space structure, and *via* polarization manipulation, a new geometric phase known as the Pancharatnam–Berry phase was created. The use of subwavelength gratings in conjunction with liquid crystals aids in the conversion of SAM to OAM,^24^ paving the way for the generation of optical vortices by polarization manipulation.^25–27^ Subsequently, researchers have reported various optical vortex-generation techniques and advances in the field, including spatial light modulators,^28,29^ OAM mode array generation by Dammann grating,^30,31^ phase only diffractive optical elements,^32–34^ spiral phase plates,^35–38^ the fabrication of various vortex-generating structures by femtosecond laser direct writing,^39,40^ the achievement of phase discontinuity by utilizing subwavelength metallic nano-antennas, scatterers, or thin films,^41–43^ Indeed, improving the vortex-generating methods has been a continuous research effort in the optical and plasmonic research societies for the past 30 years.

Plasmonic lenses have been proven to be an effective way of coupling electrons with electromagnetic waves, like light, ultimately giving rise to surface plasmon polaritons (SPPs). In a pivotal study by Liu *et al.*^44^ in 2005, the concentration of an electromagnetic field was experimentally and theoretically demonstrated through the interference of propagating SPP waves in circular and elliptical slit structures. The exploration of symmetrical structures, like circles or ellipses, has led researchers to investigate constructive and destructive interference by varying certain parameters, such as the circle's radius or the ellipse's eccentricity at specific wavelengths,^45^ as well as the detection of the OAM of incident light.^46^ Asymmetric structures, like Archimedes' spiral lens, were first studied theoretically and experimentally by Ohno and Miyanishi^47^ in 2006, in a study where they showed that the topological charge of SPPs depend on both the chirality of the spiral structure and the incident beam. Spiral slit structures have also been investigated in studies of circular polarization analyzers,^48^ the spin dependence of surface confined plasmonic waves,^49^ and the manipulation of the OAM of plasmonic waves by increasing the number of turns of the plasmonic lens,^50^*etc.* To introduce an additional degree of freedom in controlling the geometrical topological charge of plasmonic vortices, Archimedes' spiral lenses were split to create a new type of lens called the plasmonic vortex lens (PVL).^51–55^ Further modifications at the nano-metre level were later made using plasmonic metasurfaces or meta lenses to control the geometrical charges of plasmonic waves.^56–59^ Srivastava *et al.*^60^ initially conceived the concept of a hexagonal lens inscribed on topological insulator surface states. Their observations indicated an increase in the number of optical vortices within the hexagonal plasmonic lens compared to a circular lens structure. Additionally, the hexagonal lens exhibited scaling behaviour concerning changes in the lens radius and incident wavelength. The uniqueness of the hexagonal lens lies in its capability to multiply the number of optical vortices, a phenomenon not observed in the aforementioned lens structures. However, a limitation in their work was the absence of an explanation for the selection of the hexagonal structure over other possible polygonal structures. This gap served as the motivation and starting point for our work. Our objective in this study was to determine the optimal polygonal structure by conducting a theoretical and numerical comparison of various polygonal structures.

It was observed that in a hexagonal lens, there is only a single vortex at the centre, surrounding a 2nd layer of 6 vortices, a 3rd layer of 12 vortices, and so on. As we increase the lens radius, new layers of optical singularity points keep on adding. So, it is imperative to think that if we make an octagonal lens, we would obtain layers of optical vortices, such as, 1, 8, 16 or in this progression. If the sides of the lens are further increased to 10, 12, *etc.*, we should see more optical vortices. However, contrary to the expectation, in this work, we found that the number of vortices did not increase with the increment in the number of sides of the polygon. With the number of the sides of the polygon increasing, the number of optical vortices actually decreased. Then we shifted our attention to check whether one can get more vortices by reducing the number of sides of the polygon to a pentagonal and square lens. While in the case of the square lens, it seemed like the number of vortices was increasing, they seemed hugely superimposed and it was sometimes difficult to identify and distinguish the singularity points in the vortices. These observations bring us back to our initial assumption that a hexagonal structure produces the maximum number of prominent optical vortices and optical singularity points in both right-circular polarized (RCP) and left-circular polarized (LCP) illumination. In order to understand the relationship between polygons and the generation of optical vortices, in this study, we carried out detailed theoretical calculations, which were further validated by finite-difference time-domain (FDTD) simulations. Herein, we posited a unified equation to explain the generation of optical vortices in any polygonal plasmonic lens under RCP and LCP illumination. To the best of our knowledge, there are no reports in the literature providing such a kind of mathematical formulation for polygonal plasmonic lenses. Our work potentially opens up new avenues of research for polygonal plasmonic lens similar to spiral lens, plasmonic vortex lens, and meta-lens. We also switched from the widely utilized plasmonic materials, such as gold and silver, to Weyl semimetals, owing to the improved performance metric, *i.e.* the plasmonic figure of merit (FOM) of Weyl semimetals at higher frequencies. However, our unified mathematical formulation should be valid for any material, including Au and Ag.

## Material selection

**Fig. 1:**
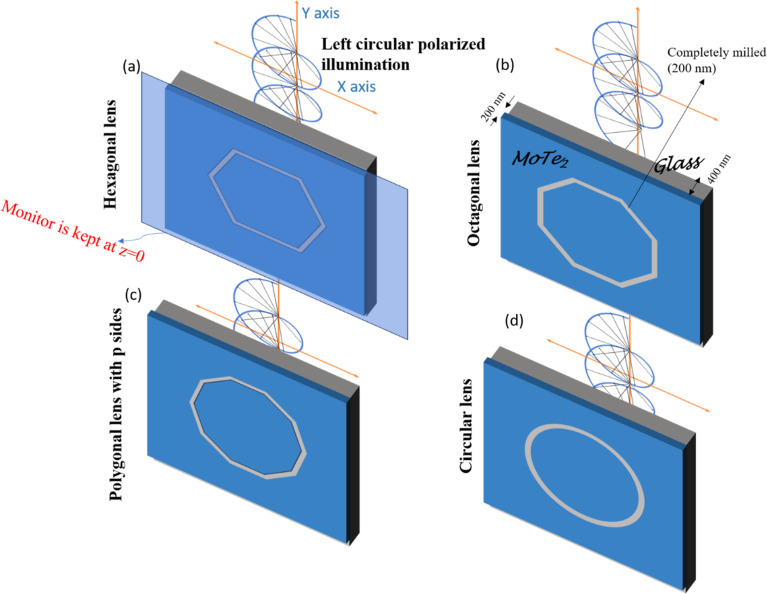
(a) Isometric view of a hexagonal lens, (b) octagonal lens, (c) polygonal lens with the number of sides *p*, and (d) circular lens.

Weyl semimetals outperform the popular plasmonic metals, such as gold and silver, in terms of plasmonic figure of merit (FOM) and surface plasmon polariton wavelength (*λ*_SPP_). The *λ*_SPP_ and FOM of any plasmonic material can be expressed mathematically as,1
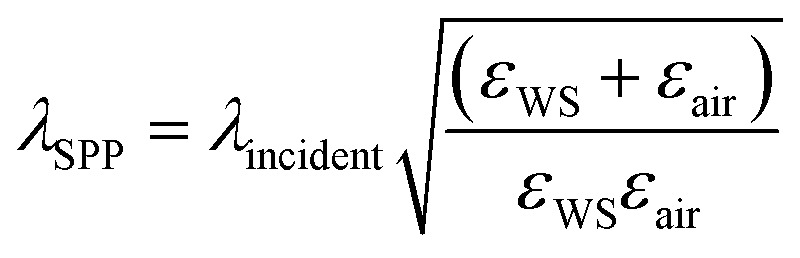
where, *ε*_WS_ or 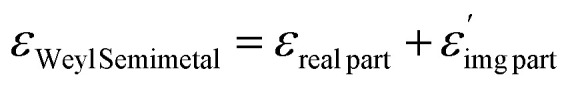
2
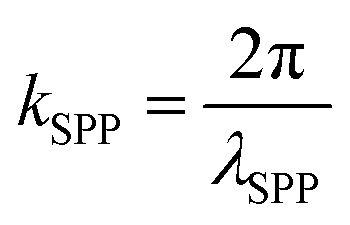
3
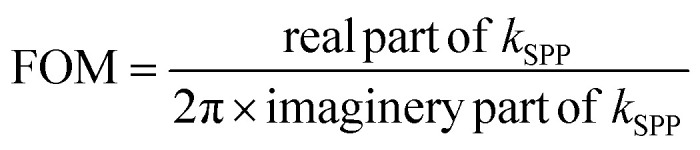
where *ε*_WS_ is the permittivity of a Weyl semimetal, *ε*_air_ is the permittivity of air, and *λ*_incident_ is the incident wavelength of the illumination. The permittivity values of MoTe_2_ were taken from experimental data.^61^ The FOM of any material represents the number of waves that surface plasmons can possibly travel, and *λ*_SPP_ represents the wavelength of the surface plasmons due to the effect of illuminating light. The comparison of the FOMs between gold, silver, and MoTe_2_ Weyl semimetal, as shown in Fig. S1 of the ESI,† infers that MoTe_2_ offers a better FOM, especially at higher frequencies. Further insights on Weyl semimetal surface states are also provided in ESI S1.† The *λ*_SPP_ values of MoTe_2_ at three commonly used incident wavelengths of 350, 375, and 415 nm were estimated to be 352.8, 379.39, and 419.85 nm, respectively. Here, we designed various plasmonic lens structures milled on MoTe_2_ through a silica substrate. A 200 nm thick MoTe_2_ Weyl semimetal layer was deposited on top of a 400 nm thick silica layer, as shown in Fig. 1. Different lens structures were entirely etched with a thickness of 200 nm. Although only LCP illumination is shown in the schematic, both RCP and LCP illumination were also used in the simulation. Fig. 1a–c show the hexagonal, octagonal, and polygonal lens with sides *p*. When the number of sides was increased to infinity, a circular structure was obtained, as shown in Fig. 1d. The design parameters were maintained for all the polygonal lens structures. Although the thickness values of Au and MoTe_2_ are schematically shown in Fig. 1 only for the octagonal lens, the same thickness was maintained for all the other polygonal structures. Additionally, the difference between the inner and outer radii of the lens structures was kept as 200 nm. The electrical field intensity and phase were measured in the near field, so the monitor was kept at the *z* = 0 position. Theoretical calculations were also performed in this order, as detailed in the next section.

### Theoretical calculations of the electrical field and phase equations of different polygonal lens structures

We started our calculation by deriving the electric field distribution in the *z*-direction (*E*_*z*_) in the centre of a hexagonal plasmonic lens by changing the equations of the circular plasmonic lens accordingly. For SPPs, the *z*-direction electric field intensities will be the most prominent factor, therefore the focus was directed only on the *E*_*z*_ component. The corresponding equation of an electric field of a circularly polarized lens (CPL) at an observation point (*ρ*, *θ*) along an incremental length in the *z*-direction of a slit^57^ can be given as:

4 d*E*_*z*_(*ρ*, *θ*, *z*) = *A*(*ϕ*(*θ*′))*e*^−*k*_a_*z*^*e*^*ω*(*ϕ*(*θ*′),*θ*′)^*e*^*jk*_spp_|*ρ*−*ρ*′|^d*θ*′

where *ρ*, *θ*, and *z* are the radial, azimuthal and *z*-direction co-ordinates of *E*_*z*_ (d*E*_*z*_ = differential form of *E*_*z*_) at the observation point, (*ρ*′, *θ*′) are the co-ordinates of the dipole source, *A*(*ϕ*(*θ*′)) and *ω*(*ϕ* (*θ*′), *θ*′) are the amplitude and phase profile functions at each dipole source, respectively, and *k*_a_ is the attenuation coefficient in the *z*-direction of the SPP mode in the air. Since, we are considering the output monitor at the surfaces of the lens, we took the attenuation coefficient as 1 and because *z* = 0, the term *e*^−*k*_a_*z*^ becomes 1. Furthermore, *j* is a complex entity, |*ρ* − *ρ*′| represents the distance between the source of the SPPs and the point of investigation, *ϕ*(*θ*′) is an azimuthal distribution function of dipole orientations with respect to the *x*-axis; or *φ*(*θ*′) is azimuthal distribution function of the dipole orientations with respect to the radial vector, *i.e. φ*(*θ*′) = *ϕ*(*θ*′) − *θ*′; and *k*_SPP_ is the SPP wave number.

The electric field component of surface plasmons^62^ propagating along the *x*-axis can be written as:5
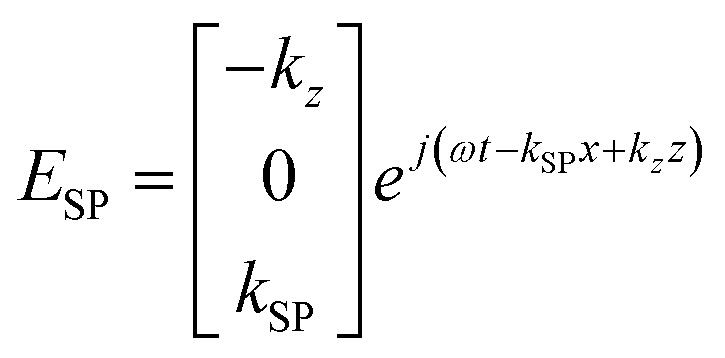


The wave vector *k*_*z*_ depicts the intensity decay along the *z*-direction and *k*_SP_ surface plasmon polariton wave vector. As *k*_*Z*_ ≪ *k*_SP_, the z-component will dominate in the near field. This is why we are only interested in the *z*-component of the electric field.

Eqn (4), the equation for a circularly polarized lens (CPL), was modified by applying some boundary conditions as shown below.*A*(*ϕ*(*θ*′)) = *A*_0_cos*ϕ*(*θ*′);*ω*(*ϕ*(*θ*′), *θ*′) = ±*ϕ*(*θ*′)Here, the +ve and −ve signs represents LCP and RCP, respectively.

For circular polarization, the dipole sources are aligned parallel to the radius, so *φ*(*θ*′) = 0 and *ϕ*(*θ*′) = *θ*′

In the case of RCP polarization:*ω*(*ϕ*(*θ*′), *θ*′) = −*ϕ*(*θ*′) = −*θ*′ and *A*(*ϕ*(*θ*′)) = *A*_0_cos*φ*(*θ*′) = *A*_0_.

The distance between the point of investigation and the source of plasmons can be represented in a vector form as 
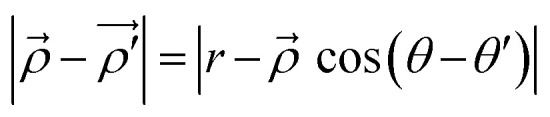
 and its modulus can be found out using the cosine rule as,


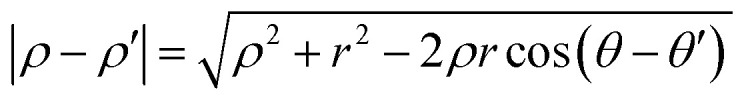
, where *r* is the radius of the circle.

Now, plugging all the values in to eqn (4) and integrating d*E*_*z*_ over 0 to 2π, we get,6
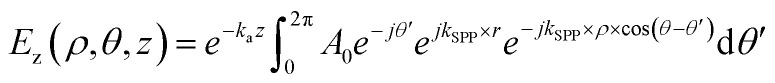


**Fig. 2:**
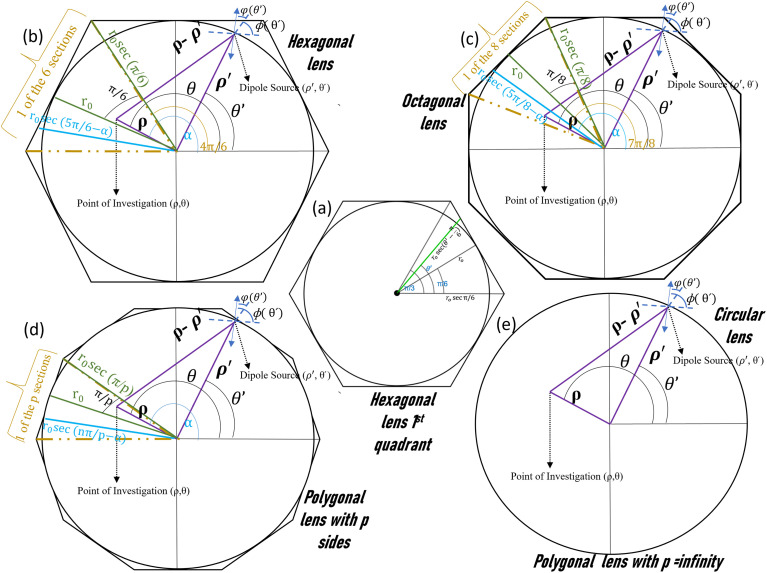
Schematic representation of the electric field derivations of a (b) hexagonal lens, (c) octagonal lens, (d) polygonal lens with *p* number of sides, and (e) circular lens. (a) Represents the modifications of electric field equations in the first quadrant of the hexagonal lens through the properties of a triangle.

Using the modulus of distance by applying the cosine rule, eqn (6) can be written as,7
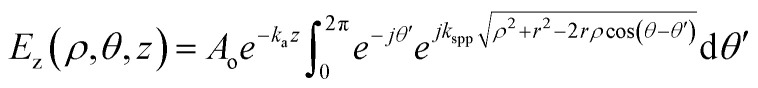
where *r* follows the relation, 
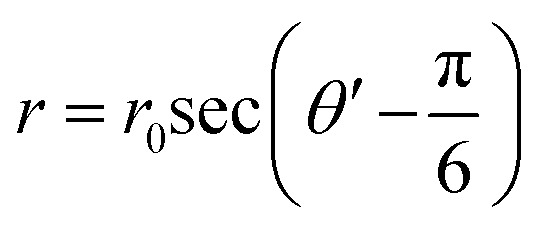
 in the 1st quadrant for a hexagonal plasmonic lens (HPL), as shown in Fig. 2a. If we rotate this line, *i.e.* the radius of the hexagon starting from the angle 0° to 2π, it touches the circle 6 times, at the angles of π/6, 3π/6, 5π/6, 7π/6, 9π/6, and 11π/6 and at these angles *r* becomes *r*_0_, which is also evident from the relationship between *r* and *r*_0_. By putting 
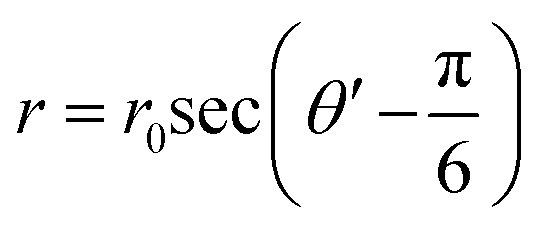
, the electric field for the range 
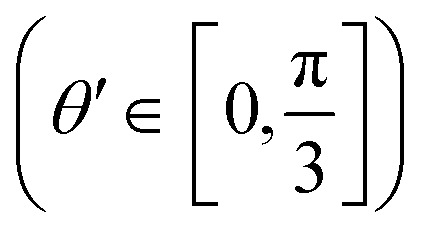
 can be represented as per Fig. 2a and as below,8
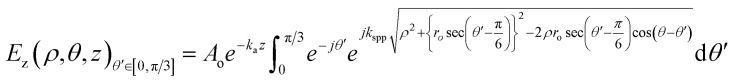


As this is a regular hexagon, we can apply almost the same equation for the remaining sections, with the integration limit *θ*′ ∈ [π/3, 2π/3], [2π/3, π], [π, 4π/3], [4π/3, 5π/3], [5π/3, 2π]. For the total hexagonal lens structure, the total electric field can thus be written as shown in Fig. 2b and as below,9
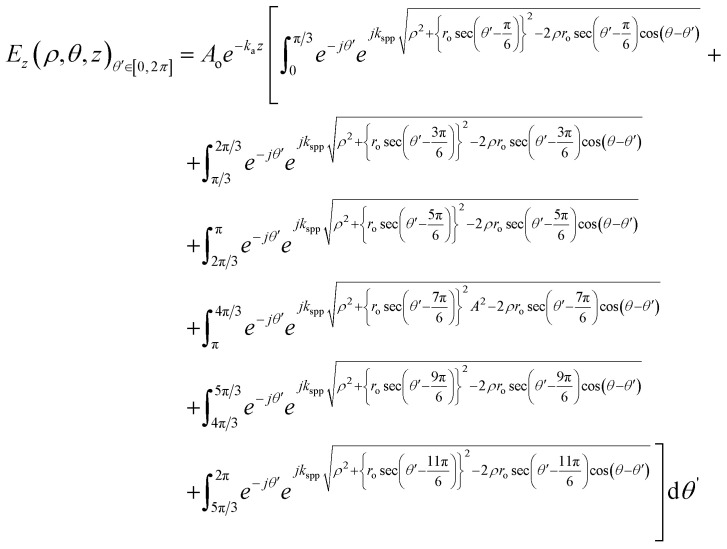


The square of eqn (9) yields the intensity, as *I* = *E*_*z*_^2^. We solved the equations in MATLAB using Simpson's 3/8th rule. The phase distribution and intensity were guided by two governing parameters: the change of *r*_0_ and *K*_SPP_. The analytical plots, obtained from MATLAB, matched well with the FDTD simulations, as discussed in the following section.

In the case of LCP illumination,*ω*(*ϕ*(*θ*′), *θ*′) = *ϕ*(*θ*′) = *θ*′.

The value of the electric field intensity at the LCP, understandable from Fig. 2b, was derived as:10
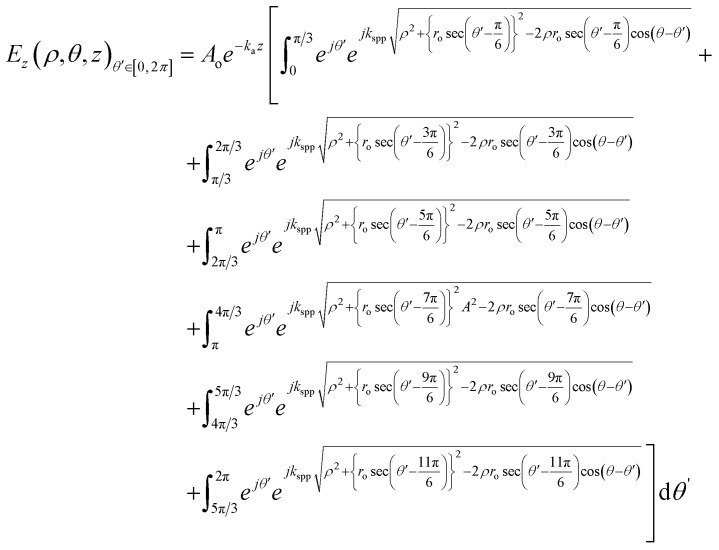


The equations for the electric field intensity arising due to the linear polarizations (*x*-polarized and *y*-polarized illumination) are discussed in the ESI S2.† From eqn (9) and (10), we propose a generalized formula for any polygonal structure having the number of sides *p* for RCP and LCP:11
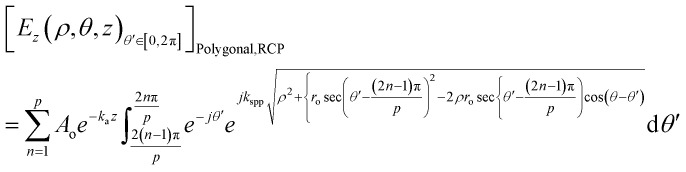
12
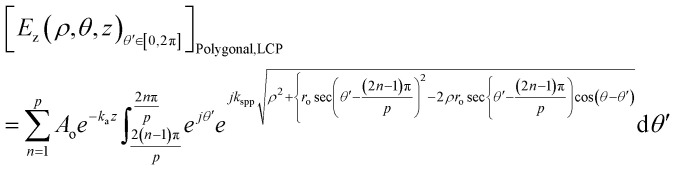
where *p* is the number of sides of the polygonal.

The unified equations for the polygonal structures (eqn (11) and (12)) clearly indicate that the singularity points are dependent on the material's properties and incident wavelength, as *k*_spp_ = *f*(*ε*, *ε*′, *λ*). These points are also influenced by the number of sides of the polygon (*p*) and the radius of the lens (*r*_o_). These key takeaways from the unified equation offer a flexibility for microparticle manipulation and vortex generation by adjusting these parameters.

The equations for heptagonal (*p* = 7) lens and octagonal (*p* = 8) lens were derived from eqn (11) and (12) and are stated in the ESI S3 and S4.† The generalized formula for a polygonal structure under linearly polarised illumination is provided in the ESI S5.†

When we keep on increasing the number of sides of the polygonal, eventually it becomes a circle for *p* = ∞ and we have proven that the generalized equation (eqn (12)) postulated for a polygonal structure indeed works for a circular lens too.

In eqn (12), when *p* tends to infinity, the 
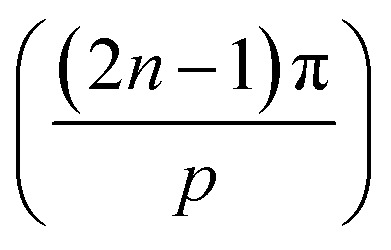
 term tends to become *θ*′ for every *n*. So, for every *n*, the 
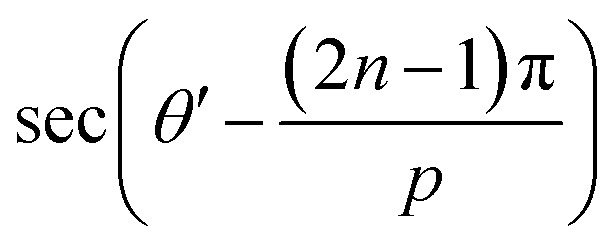
 term becomes 0. For *n* starting from 1 and going up to a high number and the 
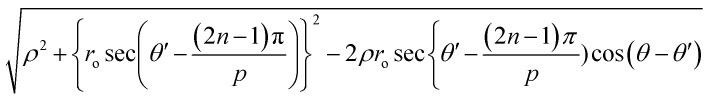
 term remains the same, this gets converted to 
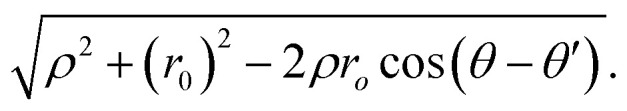


So, when *p* is infinity, eqn (12) can be written as:13
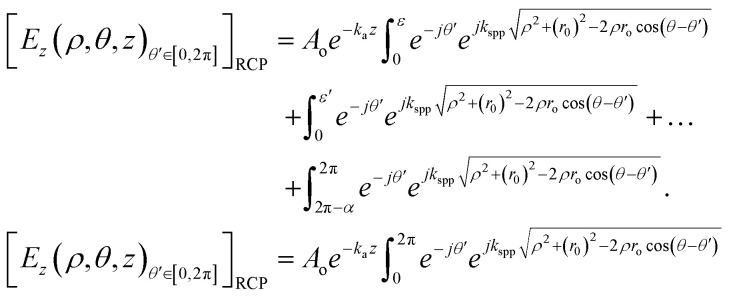
14
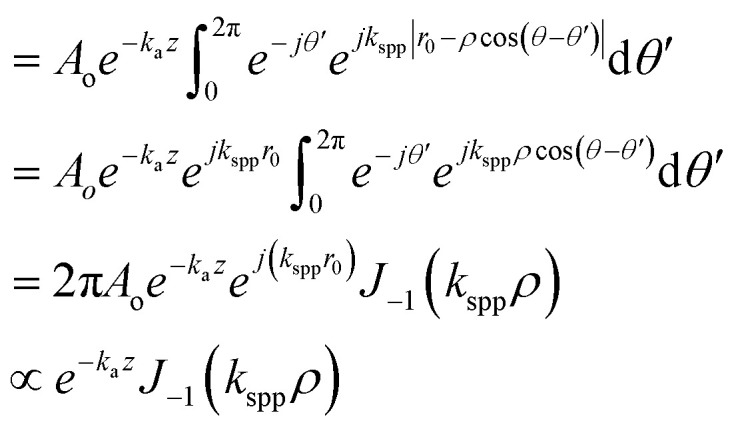


Similarly, for LCP, the electric field will be:15
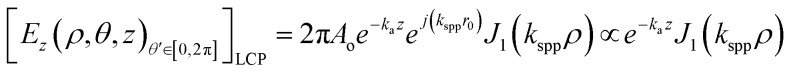


### Comparison of different polygonal plasmonic lens structures by FDTD simulation and theoretical calculations

In the previous section, we derived equations for the electric field intensity and phase distribution of various plasmonic lens structures. In this section, the electric field intensity at *z*-direction and phase distribution have been simulated in FDTD and also have been plotted in MATLAB based on the theoretical calculations.

**Fig. 3:**
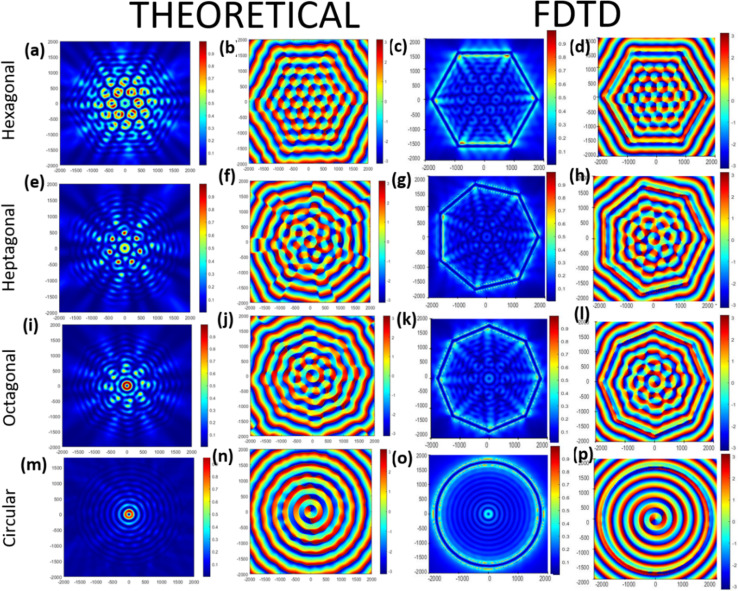
MATLAB plots and FDTD simulations of the intensity and phase distribution of different lens structures, *i.e.*, hexagonal lens (a, b, c, and d), heptagonal lens (e, f, g, and h), octagonal lens (i, j, k, and l), and circular lens (m, n, o, and p) under RCP illumination.

We started our investigation by carrying out FDTD simulations for various plasmonic lenses with different lens geometries, namely hexagonal, heptagonal, octagonal, and circular lenses. For all the plasmonic lens structures, the radii of the lenses were taken as *r* = 5 *λ*_spp_. For the hexagonal lens under RCP illumination, as shown in Fig. 3c and d, we could observe a single optical vortex in the centre surrounded by the 2nd layer of 6 optical vortices and the 3rd layer of 12 optical vortices. It is anticipated that one can obtain more optical vortices, if we keep on increasing the radius of the plasmonic lens. Based on the results obtained for the hexagonal lens, *i.e.* a polygon with the number of sides = 6, one can possibly expect similar increasing number of optical vortices to be formed layer-by-layer in the case of an octagonal lens, *i.e.* a polygon with *n* = 8. However, in our FDTD simulation and theoretical calculations, the number of vortices appearing for the octagonal lens with the same radius was much lower than for the hexagonal lens, since only the 1st and 2nd layers of vortices were formed for an octagonal lens. *The* heptagonal lens also produced a singularity point in the centre despite being an asymmetric structure, while the circular lens showed only a single optical singularity point in the centre. For LCP illumination, similar results were obtained for all the lenses, as shown in the ESI Fig. S2.†

**Fig. 4:**
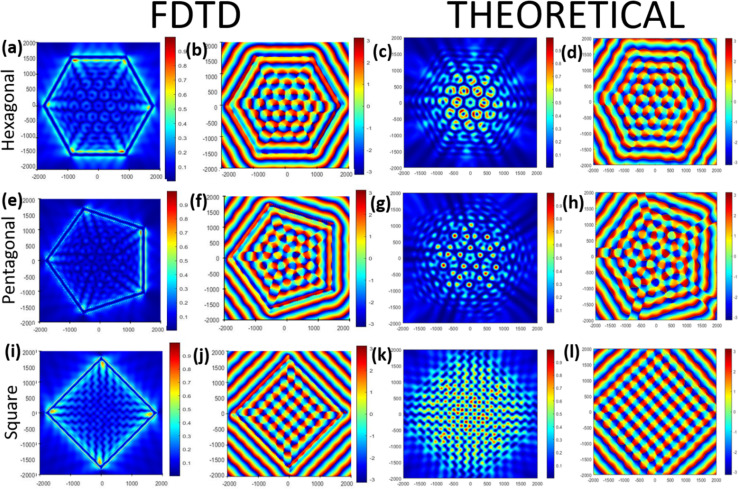
Intensity and phase distribution of different lens structures, *i.e.* hexagonal lens (a, b, c, and d), pentagonal lens (e, f, g, and h), and square-shaped lens (i, j, k, and l). 1st and 2nd columns represent the FDTD simulations and 3rd and 4th column represent the MATLAB plots for a lens radius *r*_0_ = 5*λ*_spp_ in MoTe_2_ under RCP illumination.

The MATLAB plots resembled the plots for the FDTD simulations, as shown in Fig. 3. The only difference between the two plots is in the intensity plots near or around the perimeter of the lens. This difference was probably because FDTD gives a more accurate result near the boundary of the lens, whereas in the MATLAB simulation, we just plotted the equation. The total topological charge of the vortices produced in each of the lens structures was found to be +1 or −1, depending on the nature of the illumination. LCP produced a topological charge of +1 and RCP-1, indicating that the geometrical topological charge of any lens structures is zero, irrespective of the number of sides of the polygons. From Fig. 4, it is evident that the more we increase the number of sides of the plasmonic lens, the lesser the number of plasmonic vortices, when keeping every other condition intact. This is the reason behind getting only one singular point, when the number of sides becomes infinity, *i.e.* a circular plasmonic lens. Furthermore, we investigated smaller sided structures than a hexagon. In Fig. 4, a relative comparison through colour maps between hexagonal, square-shaped, and pentagonal lens is shown only for LCP illumination.

The number of optical vortices seemed to increase for the case of the square lens compared to the hexagonal lens, but the singularity points were very difficult to differentiate from each other and seemed like a huge superimposition. These observations indicate that the hexagonal lens is the most optimized lens structure in terms of optical vortex generation. Also, the effect of linearly polarised lights on the different lens structures investigated through FDTD simulations and MATLAB plots and further insights on the theoretical investigations of singularity points are reported in the ESI S7.† Next, we further delved into the far-field studies of the best performing plasmonic lens structure, *i.e.* hexagonal lens, as shown in Fig. S6.†

**Fig. 5:**
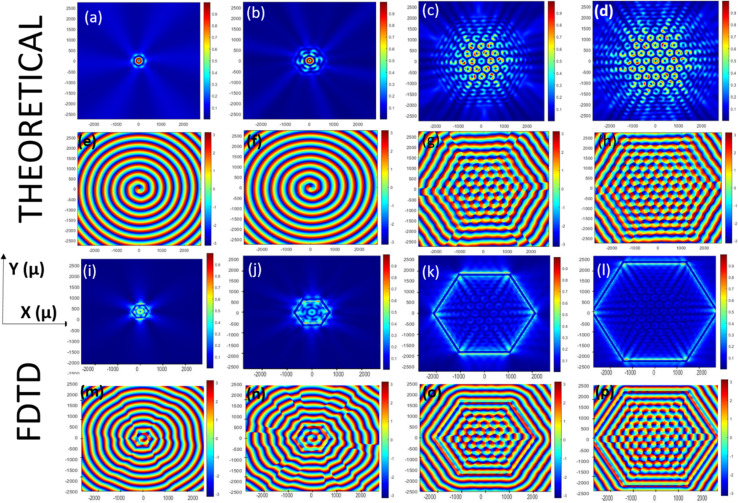
Theoretical plots [(a), (b), (c), (d), (i), (j), (k), and (l)] and FDTD simulations [(e), (f), (g), (h), (m), (n), (o), and (p)] of a hexagonal lens with varying radii of *r*_0_ = *λ*_spp,_(a, e, i, and m), 2*λ*_spp_ (b, f, j, and n), 6*λ*_spp,_ (c, g, k, and o), (d, h, l, and p) 8*λ*_spp_ for RCP at 350 nm wavelength.

We discuss the vortex multiplexing phenomenon, as a function of the size of lens structure, in the next section. The number of optical vortices increased as we increased the radius of the lens, as shown in Fig. 5. The FDTD simulation results and MATLAB plots for the theoretically derived equations are presented in Fig. 5, which represents the E-field intensity and phase patterns observed with different radii of hexagonal gratings inscribed on Weyl semimetals at *r*_0_ = *λ*_SPP_, 2*λ*_SPP_, 6*λ*_SPP_, and 8*λ*_SPP_ under RCP illumination for wavelength of 350 nm leading to *λ*_SPP_ = 352.8 nm. The number of optical vortices was 1 when the lens radius was *λ*_SPP_. Then it started to increase astronomically with the lens radius. For lens radius *r*_0_ = 2*λ*_SPP_, a new 2nd layer with the number of optical vortices as 6 started to exist and the 1st layer became very prominent. With the lens radii *r*_0_ = 6*λ*_SPP_ and 8*λ*_SPP_, the numbers of distinct vortex layers were 4 and 5 surrounded by a somewhat ambiguous optical vortex layer. The number of optical vortices formed in the *n*th layer was found to follow the empirical formula of 6(*n* − 1).

This sort of ‘scaling behaviour’ was further validated by theoretical calculations, as shown in Fig. 5m–p. It is evident that the MATLAB plots of the theoretical calculation corroborated well with the FDTD results. Such a behaviour will be extremely beneficial in memory decoding applications, where each vortex can be utilized for reading information purposes. Also, such a simplicity of scaling can make the hexagonal lens a very popular lens structure. The ‘scaling behaviour’ is also counterintuitive in some senses, because if we increase the lens radius the general intuition is that the lattice constant of the vortices will increase^63^ or only the intensity around the centre^44^ will change, keeping the number of optical vortices the same. Instead of this, we get an escalation of the number of optical vortices.

We further delved into the reasoning behind the optical singularities of different plasmonic structures from the unified mathematical equation for a polygonal lens in ESI S9.† It has been demonstrated in S9 that singularity points emerge at the centre of the lens structure, (*i.e.* at co-ordinates *ρ*, *θ* = 0, 0) and at the co-ordinates 
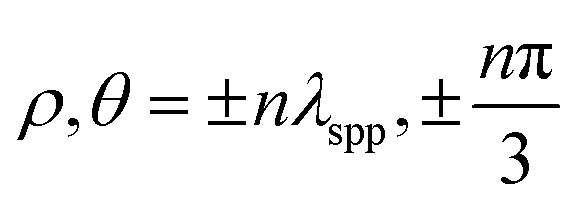
, where *n* takes any integer value starting from 1. The range of *n* is proportionally dependent on the radius of the lens structure. However, increasing the radius indefinitely is not feasible due to the limited propagation length of plasmons.

This is where Weyl semimetals, such as MoTe_2_, prove advantageous. MoTe_2_ has a higher figure of merit (FOM) than most noble metals, allowing plasmons to travel farther before their amplitude diminishes. This extended propagation distance with MoTe_2_ offers greater flexibility in increasing the radius of the lens structure compared to with noble metals like Au and Ag, which, in turn, enables the generation of a larger number of vortices.

## Conclusion

In summary, we presented here an optimized plasmonic lens structure inscribed on Weyl semimetals based on unified mathematical formulations. We further compared the colour contour plots made with MATLAB with FDTD simulations. We observed the generation of more optical vortices in a hexagonal lens than circular lens within the same etching radius span, which implies a larger area density of optical vortices in this plasmonic lens structure, followed by two counterintuitive phenomena. The first one was the increment of the number of optical vortices with the lens radius instead of the lattice constant and second, the number of optical vortices decreased when number of sides of the lens increased. With the help of the above observations and after comparing every possible polygonal lens structures, we came to the conclusion that the hexagonal lens structure is the best plasmonic lens structure in terms of optical vortices generation. Furthermore, the generalized mathematical expression proposed in this work can be used to calculate the performance of any plasmonic lens.

## Data availability

Data supporting this article have been included in the manuscript and as part of ESI.† The MATLAB codes for the mathematical equations are available from the authors upon request.

## Conflicts of interest

The authors declare that they have no known competing financial interests or personal relationships that could have appeared to influence the work reported in this paper.

## Supplementary Material

NA-OLF-D4NA00336E-s001
