# Supplementary material for: Optimization of a plasmonic lens structure for maximum optical vortices induced on Weyl semimetal surface states
Source: Nanoscale Adv. 2024 Sep 10;6(23):5960–70. doi: 10.1039/d4na00336e (PMC11446401; doi:10.1039/d4na00336e)
Supplement: NA-006-D4NA00336E-s001 [file NA-006-D4NA00336E-s001.pdf]

Supplementary Information (SI) for Nanoscale Advances.
This journal is © The Royal Society of Chemistry 2024

Optimization of plasmonic lens structure for maximum optical vortices induced on Weyl Semimetals Surface States

Ritwik Banerjee[1] and Tanmoy Maiti[11]

[1] Plasmonics and Perovskites Laboratory, Indian Institute of Technology Kanpur, UP, 208016, India

Supporting information

S1. Comparison of figure of merit (FOM) between different materials

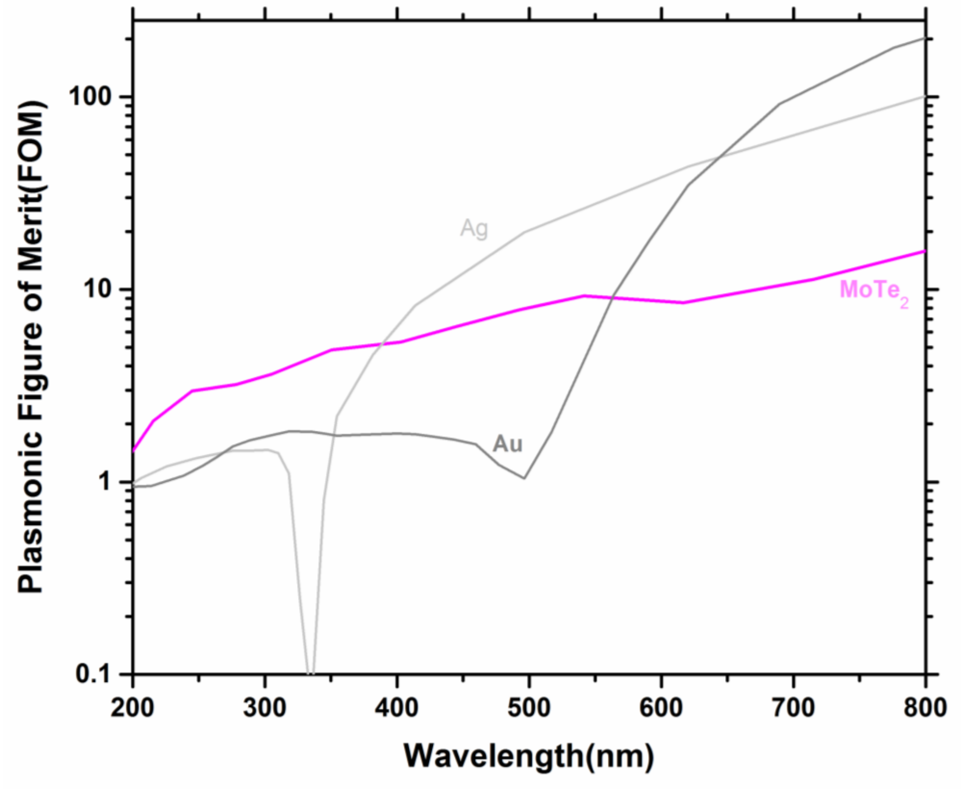

Figure S1: Comparison of figure of merits between $MoTe_2$ Weyl semimetal, Au and Ag.

Type II Weyl semimetal surface state is prominent in $MoTe_2$. [1] The permittivity matrix for Weyl semimetals surface states can be represented as:

[1] Corresponding author Tel: +91-512-259-6599
Email address: tmaiti@iitk.ac.in (Tanmoy Maiti)

$$\varepsilon_{WS} = \begin{pmatrix} \varepsilon_{xx} & \varepsilon_{xy} & \varepsilon_{xz} \\ \varepsilon_{xy}^{*} & \varepsilon_{yy} & \varepsilon_{yz} \\ \varepsilon_{xz}^{*} & \varepsilon_{yz}^{*} & \varepsilon_{zz} \end{pmatrix}$$

Here, off diagonal elements ε_{xy}^{*}, ε_{xz}^{*} and ε_{yz}^{*} represents the complex conjugate of ε_{xy}, ε_{xz} and ε_{yz}. The non-zero off-diagonal tensor elements originate from the non-vanishing Berry curvature of the Weyl semimetals.

S2. Analytical calculations of Electric field and phase maps under x-polarized and y-polarized illumination for hexagonal lens:

Further let's extend our calculation of electric field for linearly polarized lights. In case of x-polarized light, $\varphi(\theta') = \theta'$ and $(\theta') = 0$.

$\omega(\phi(\theta'), \theta') = \phi(\theta') = 0$.

$A(\phi(\theta')) = A_0 \cos\varphi(\theta') = A_0 \cos\theta'$.

Plugging all the values in equation 3 and integrating it to 0 to 2π, we get-

$$E_z(\rho, \theta, z)_{\theta' \epsilon [0,2\pi]} = A_o e^{-k_a z} \left[\int_0^{\pi/3} \cos\theta'\, e^{jk_{spp}\sqrt{\rho^2 + \{r_o \sec(\theta' - \frac{\pi}{6})\}^2 - 2\rho r_o \sec(\theta' - \frac{\pi}{6}) \cos(\theta - \theta')}} + \right.$$

$$\int_{\pi/3}^{2\pi/3} \cos\theta'\, e^{jk_{spp}\sqrt{\rho^2 + \{r_o \sec(\theta' - \frac{3\pi}{6})\}^2 - 2\rho r_o \sec(\theta' - \frac{3\pi}{6}) \cos(\theta - \theta')}} +$$

$$\int_{2\pi/3}^{\pi} \cos\theta'\, e^{jk_{spp}\sqrt{\rho^2 + \{r_o \sec(\theta' - \frac{5\pi}{6})\}^2 - 2\rho r_o \sec(\theta' - \frac{5\pi}{6}) \cos(\theta - \theta')}} +$$

$$\int_{\pi}^{4\pi/3} \cos\theta'\, e^{jk_{spp}\sqrt{\rho^2 + \{r_o \sec(\theta' - \frac{7\pi}{6})\}^2 - 2\rho r_o \sec(\theta' - \frac{7\pi}{6}) \cos(\theta - \theta')}} +$$

$$\int_{4\pi/3}^{5\pi/3} \cos\theta' \; e^{jk_{spp}\sqrt{\rho^2+\{r_o \sec(\theta'-\frac{9\pi}{6})\}^2-2\rho r_o \sec(\theta'-\frac{9\pi}{6})\cos(\theta-\theta')}} +$$

$$\left.\int_{5\pi/3}^{2\pi} \cos\theta' \, e^{jk_{spp}\sqrt{\rho^2+\{r_o \sec(\theta'-\frac{11\pi}{6})\}^2-2\rho r_o \sec(\theta'-\frac{11\pi}{6})\cos(\theta-\theta')}}\right] d\theta' \; \ldots (S1)$$

For y-polarised light, $\varphi(\theta') = \frac{\pi}{2} - \theta'$ and $(\theta') = \frac{\pi}{2}$.

$\omega(\phi(\theta'), \theta') = \phi(\theta') = {}^{\pi}/_{2}$.

$A(\phi(\theta')) = A_0 \cos\varphi(\theta') = A_0 \cos\left(\frac{\pi}{2} - \theta'\right) = A_0 \sin\theta'$.

Calculating electric field in z-direction for y-polarized light, we get-

$E_z(\rho, \theta, z)_{\theta' \epsilon [0,2\pi]}$

$$= A_o e^{-k_a z}\left[\int_{0}^{\pi/3} sin\theta' e^{jk_{spp}\sqrt{\rho^2+\{r_o \sec(\theta'-\frac{\pi}{6})\}^2-2\rho r_o \sec(\theta'-\frac{\pi}{6})\cos(\theta-\theta')}}\right.$$

$$+ \int_{\pi/3}^{2\pi/3} sin\theta' e^{jk_{spp}\sqrt{\rho^2+\{r_o \sec(\theta'-\frac{3\pi}{6})\}^2-2\rho r_o \sec(\theta'-\frac{3\pi}{6})\cos(\theta-\theta')}}$$

$$+ \int_{2\pi/3}^{\pi} sin\theta' e^{jk_{spp}\sqrt{\rho^2+\{r_o \sec(\theta'-\frac{5\pi}{6})\}^2-2\rho r_o \sec(\theta'-\frac{5\pi}{6})\cos(\theta-\theta')}}$$

$$+ \int_{\pi}^{4\pi/3} sin\theta' e^{jk_{spp}\sqrt{\rho^2+\{r_o \sec(\theta'-\frac{7\pi}{6})\}^2-2\rho r_o \sec(\theta'-\frac{7\pi}{6})\cos(\theta-\theta')}}$$

$$+ \int_{4\pi/3}^{5\pi/3} sin\theta' e^{jk_{spp}\sqrt{\rho^2+\{r_o \sec(\theta'-\frac{9\pi}{6})\}^2-2\rho r_o \sec(\theta'-\frac{9\pi}{6})\cos(\theta-\theta')}}$$

$$\left.+ \int_{5\pi/3}^{2\pi} sin\theta' e^{jk_{spp}\sqrt{\rho^2+\{r_o \sec(\theta'-\frac{11\pi}{6})\}^2-2\rho r_o \sec(\theta'-\frac{11\pi}{6})\cos(\theta-\theta')}}\right] d\theta' \; \ldots (S2)$$

$e^{-j\theta'}$ can be written as $(\cos\theta' - \mathrm{j}\sin\theta')$. Therefore, equation 9 in the main manuscript, electric field intensity for hexagonal lens, under RCP illumination can also be written as-

$E_z(\rho,\theta,z)_{\,\theta'\epsilon[0,2\pi]} =$

$$A_o e^{-k_a z}\left[\left[\left\{\int_0^{\pi/3} \cos\theta'\, e^{jk_{spp}\sqrt{\rho^2+\{r_o\sec(\theta'-\frac{\pi}{6})\}^2-2\rho r_o\sec(\theta'-\frac{\pi}{6})\cos(\theta-\theta')}} +\right.\right.\right.$$

$$\int_{\pi/3}^{2\pi/3} \cos\theta'\, e^{jk_{spp}\sqrt{\rho^2+\{r_o\sec(\theta'-\frac{3\pi}{6})\}^2-2\rho r_o\sec(\theta'-\frac{3\pi}{6})\cos(\theta-\theta')}} +$$

$$\int_{2\pi/3}^{\pi} \cos\theta'\, e^{jk_{spp}\sqrt{\rho^2+\{r_o\sec(\theta'-\frac{5\pi}{6})\}^2-2\rho r_o\sec(\theta'-\frac{5\pi}{6})\cos(\theta-\theta')}} +$$

$$\int_{\pi}^{4\pi/3} \cos\theta'\, e^{jk_{spp}\sqrt{\rho^2+\{r_o\sec\left(\theta'-\frac{7\pi}{6}\right)\}^2-2\rho r_o\sec\left(\theta'-\frac{7\pi}{6}\right)\cos(\theta-\theta')}} +$$

$$\int_{4\pi/3}^{5\pi/3} \cos\theta'\, e^{jk_{spp}\sqrt{\rho^2+\{r_o\sec(\theta'-\frac{9\pi}{6})\}^2-2\rho r_o\sec(\theta'-\frac{9\pi}{6})\cos(\theta-\theta')}} +$$

$$\left.\int_{5\pi/3}^{2\pi} \cos\theta'\, e^{jk_{spp}\sqrt{\rho^2+\{r_o\sec(\theta'-\frac{11\pi}{6})\}^2-2\rho r_o\sec(\theta'-\frac{11\pi}{6})\cos(\theta-\theta')}}\right\} -$$

$$j\left\{\int_0^{\pi/3} sin\theta' e^{jk_{spp}\sqrt{\rho^2+\{r_o\sec(\theta'-\frac{\pi}{6})\}^2-2\rho r_o\sec(\theta'-\frac{\pi}{6})\cos(\theta-\theta')}} +\right.$$

$$\int_{\pi/3}^{2\pi/3} sin\theta' e^{jk_{spp}\sqrt{\rho^2+\{r_o\sec(\theta'-\frac{3\pi}{6})\}^2-2\rho r_o\sec(\theta'-\frac{3\pi}{6})\cos(\theta-\theta')}} +$$

$$\int_{2\pi/3}^{\pi} sin\theta' e^{jk_{spp}\sqrt{\rho^2+\{r_o\sec(\theta'-\frac{5\pi}{6})\}^2-2\rho r_o\sec(\theta'-\frac{5\pi}{6})\cos(\theta-\theta')}} +$$

$$\int_{\pi}^{4\pi/3} sin\theta' e^{jk_{spp}\sqrt{\rho^2+\{r_o \sec(\theta'-\frac{7\pi}{6})\}^2-2\rho r_o \sec(\theta'-\frac{7\pi}{6})\cos(\theta-\theta')}} +$$

$$\int_{4\pi/3}^{5\pi/3} sin\theta' e^{jk_{spp}\sqrt{\rho^2+\{r_o \sec(\theta'-\frac{9\pi}{6})\}^2-2\rho r_o \sec(\theta'-\frac{9\pi}{6})\cos(\theta-\theta')}} +$$

$$\left.\left.\int_{5\pi/3}^{2\pi} sin\theta' e^{jk_{spp}\sqrt{\rho^2+\{r_o \sec(\theta'-\frac{11\pi}{6})\}^2-2\rho r_o \sec(\theta'-\frac{11\pi}{6})\cos(\theta-\theta')}}\right\}\right] d\theta' \ldots (S3)$$

So, for hexagonal lens structures electric field under RCP polarization ($E_z(\rho,\theta,z)_{RCP}$) will be a combination of electric field due to x-polarized light ($E_z(\rho,\theta,z)_{x-P}$) and y polarized light $(E_z(\rho,\theta,z)_{y-P}$ and can be written as, $E_z(\rho,\theta,z)_{RCP} = E_z(\rho,\theta,z)_{x-P} - j\,E_z(\rho,\theta,z)_{y-P}$. Similarly electric field under LCP, $E_z(\rho,\theta,z)_{LCP} = E_z(\rho,\theta,z)_{x-P} + j\,E_z(\rho,\theta,z)_{y-P}$.

S3. Analytical calculations for heptagonal lens under circular polarization:

For non-symmetric Heptagonal plasmonic lens, the electric field for LCP similarly can be derived as follows-

$$[E_z(\rho,\theta,z)_{\,\theta'\epsilon[0,2\pi]}]_{LCP} =$$

$$A_o e^{-k_a z}\left[\int_{0}^{2\pi/7} e^{j\theta'} e^{jk_{spp}\sqrt{\rho^2+\{r_o \sec(\theta'-\frac{\pi}{7})\}^2-2\rho r_o \sec(\theta'-\frac{\pi}{7})\cos(\theta-\theta')}} +\right.$$

$$\int_{2\pi/7}^{4\pi/7} e^{j\theta'} e^{jk_{spp}\sqrt{\rho^2+\{r_o \sec(\theta'-\frac{3\pi}{7})\}^2-2\rho r_o \sec(\theta'-\frac{3\pi}{7})\cos(\theta-\theta')}} +$$

$$\int_{4\pi/7}^{6\pi/7} e^{j\theta'} e^{jk_{spp}\sqrt{\rho^2+\{r_o \sec(\theta'-\frac{5\pi}{7})\}^2-2\rho r_o \sec(\theta'-\frac{5\pi}{7})\cos(\theta-\theta')}} +$$

$$\int_{6\pi/7}^{8\pi/7} e^{j\theta'} e^{jk_{spp}\sqrt{\rho^2+\{r_o \sec(\theta'-\frac{7\pi}{7})\}^2-2\rho r_o \sec(\theta'-\frac{7\pi}{7})\cos(\theta-\theta')}} +$$

$$\int_{8\pi/7}^{10\pi/7} e^{j\theta'} e^{jk_{spp}\sqrt{\rho^2+\{r_o\sec(\theta'-\frac{9\pi}{7})\}^2-2\rho r_o\sec(\theta'-\frac{9\pi}{7})\cos(\theta-\theta')}} +$$

$$\int_{10\pi/7}^{12\pi/7} e^{j\theta'} e^{jk_{spp}\sqrt{\rho^2+\{r_o\sec(\theta'-\frac{11\pi}{7})\}^2-2\rho r_o\sec(\theta'-\frac{11\pi}{7})\cos(\theta-\theta')}} +$$

$$\left.\int_{12\pi/7}^{2\pi} e^{j\theta'} e^{jk_{spp}\sqrt{\rho^2+\{r_o\sec(\theta'-\frac{13\pi}{7})\}^2-2\rho r_o\sec(\theta'-\frac{13\pi}{7})\cos(\theta-\theta')}}\right] d\theta' \ldots (S4)$$

S4. Analytical calculations for octagonal lens under circular polarization:

From equation 10 derived in the main manuscript, Electric field under RCP polarisation for octagonal plasmonic lens can also be written as-

$$E_z(\rho,\theta,z)_{\theta'\epsilon[0,2\pi]} = A_o e^{-k_a z}\left[\int_0^{\pi/4} e^{-j\theta'} e^{jk_{spp}\sqrt{\rho^2+\{r_o\sec(\theta'-\frac{\pi}{8})\}^2-2\rho r_o\sec(\theta'-\frac{\pi}{8})\cos(\theta-\theta')}} +\right.$$

$$\int_{\pi/4}^{\pi/2} e^{-j\theta'} e^{jk_{spp}\sqrt{\rho^2+\{r_o\sec(\theta'-\frac{3\pi}{8})\}^2-2\rho r_o\sec(\theta'-\frac{3\pi}{8})\cos(\theta-\theta')}} +$$

$$\int_{\pi/2}^{3\pi/4} e^{-j\theta'} e^{jk_{spp}\sqrt{\rho^2+\{r_o\sec(\theta'-\frac{5\pi}{8})\}^2-2\rho r_o\sec(\theta'-\frac{5\pi}{8})\cos(\theta-\theta')}} +$$

$$\int_{3\pi/4}^{\pi} e^{-j\theta'} e^{jk_{spp}\sqrt{\rho^2+\{r_o\sec(\theta'-\frac{7\pi}{8})\}^2-2\rho r_o\sec(\theta'-\frac{7\pi}{8})\cos(\theta-\theta')}} +$$

$$\int_{\pi}^{5\pi/4} e^{-j\theta'} e^{jk_{spp}\sqrt{\rho^2+\{r_o\sec(\theta'-\frac{9\pi}{8})\}^2-2\rho r_o\sec(\theta'-\frac{9\pi}{8})\cos(\theta-\theta')}} +$$

$$\int_{5\pi/4}^{3\pi/2} e^{-j\theta'} e^{jk_{spp}\sqrt{\rho^2+\{r_o\sec(\theta'-\frac{11\pi}{8})\}^2-2\rho r_o\sec(\theta'-\frac{11\pi}{8})\cos(\theta-\theta')}} +$$

$$\int_{3\pi/2}^{7\pi/4} e^{-j\theta'} e^{jk_{spp}\sqrt{\rho^2+\{r_o \sec(\theta'-\frac{13\pi}{8})\}^2-2\rho r_o \sec(\theta'-\frac{13\pi}{8})\cos(\theta-\theta')}} +$$

$$\left.\int_{7\pi/4}^{2\pi} e^{-j\theta'} e^{jk_{spp}\sqrt{\rho^2+\{r_o \sec(\theta'-\frac{15\pi}{8})\}^2-2\rho r_o \sec(\theta'-\frac{15\pi}{8})\cos(\theta-\theta')}}\right] d\theta' \ldots (S5)$$

Similarly for octagonal lens, Electric field due to LCP can be found out as-

$$E_z(\rho,\theta,z)_{\theta'\epsilon[0,2\pi]} = A_o e^{-k_a z}\left[\int_0^{\pi/4} e^{j\theta'} e^{jk_{spp}\sqrt{\rho^2+\{r_o \sec(\theta'-\frac{\pi}{8})\}^2-2\rho r_o \sec(\theta'-\frac{\pi}{8})\cos(\theta-\theta')}} +\right.$$

$$\int_{\pi/4}^{\pi/2} e^{j\theta'} e^{jk_{spp}\sqrt{\rho^2+\{r_o \sec(\theta'-\frac{3\pi}{8})\}^2-2\rho r_o \sec(\theta'-\frac{3\pi}{8})\cos(\theta-\theta')}} +$$

$$\int_{\pi/2}^{3\pi/4} e^{j\theta'} e^{jk_{spp}\sqrt{\rho^2+\{r_o \sec(\theta'-\frac{5\pi}{8})\}^2-2\rho r_o \sec(\theta'-\frac{5\pi}{8})\cos(\theta-\theta')}} +$$

$$\int_{3\pi/4}^{\pi} e^{j\theta'} e^{jk_{spp}\sqrt{\rho^2+\{r_o \sec(\theta'-\frac{7\pi}{8})\}^2-2\rho r_o \sec(\theta'-\frac{7\pi}{8})\cos(\theta-\theta')}} +$$

$$\int_{\pi}^{5\pi/4} e^{j\theta'} e^{jk_{spp}\sqrt{\rho^2+\{r_o \sec(\theta'-\frac{9\pi}{8})\}^2-2\rho r_o \sec(\theta'-\frac{9\pi}{8})\cos(\theta-\theta')}} +$$

$$\int_{5\pi/4}^{3\pi/2} e^{j\theta'} e^{jk_{spp}\sqrt{\rho^2+\{r_o \sec(\theta'-\frac{11\pi}{8})\}^2-2\rho r_o \sec(\theta'-\frac{11\pi}{8})\cos(\theta-\theta')}} +$$

$$\int_{3\pi/2}^{7\pi/4} e^{j\theta'} e^{jk_{spp}\sqrt{\rho^2+\{r_o \sec(\theta'-\frac{13\pi}{8})\}^2-2\rho r_o \sec(\theta'-\frac{13\pi}{8})\cos(\theta-\theta')}} +$$

$$\left.\int_{7\pi/4}^{2\pi} e^{j\theta'} e^{jk_{spp}\sqrt{\rho^2+\{r_o \sec(\theta'-\frac{15\pi}{8})\}^2-2\rho r_o \sec(\theta'-\frac{15\pi}{8})\cos(\theta-\theta')}}\right] d\theta' \ldots (S6)$$

S5. Generalized equation of electric field for polygonal lens with sides p under linear polarisation (x-P and y-P):

$$[E_z(\rho,\theta,z)_{\theta'\epsilon[0,2\pi]}]_{Polygonal,xP}$$

$$= \sum_{n=1}^{p} A_o e^{-k_a z} \int_{\frac{2(n-1)\pi}{p}}^{\frac{2n\pi}{p}} cos\theta \; e^{jk_{spp}\sqrt{\rho^2+\{r_o \sec\left(\theta'-\frac{(2n-1)\pi}{p}\right)^2 - 2\rho r_o \sec\left\{\theta'-\frac{(2n-1)\pi}{p}\right)\cos(\theta-\theta')}} d\theta' \ldots (S7)$$

$$[E_z(\rho,\theta,z)_{\theta'\epsilon[0,2\pi]}]_{Polygonal,yP}$$

$$= \sum_{n=1}^{p} A_o e^{-k_a z} \int_{\frac{2(n-1)\pi}{p}}^{\frac{2n\pi}{p}} sin\theta \; e^{jk_{spp}\sqrt{\rho^2+\{r_o \sec\left(\theta'-\frac{(2n-1)\pi}{p}\right)^2 - 2\rho r_o \sec\left\{\theta'-\frac{(2n-1)\pi}{p}\right)\cos(\theta-\theta')}} d\theta' \ldots (S8)$$

S6. MATLAB and FDTD plots of Electric field and phase of different plasmonic lens structure under LCP:

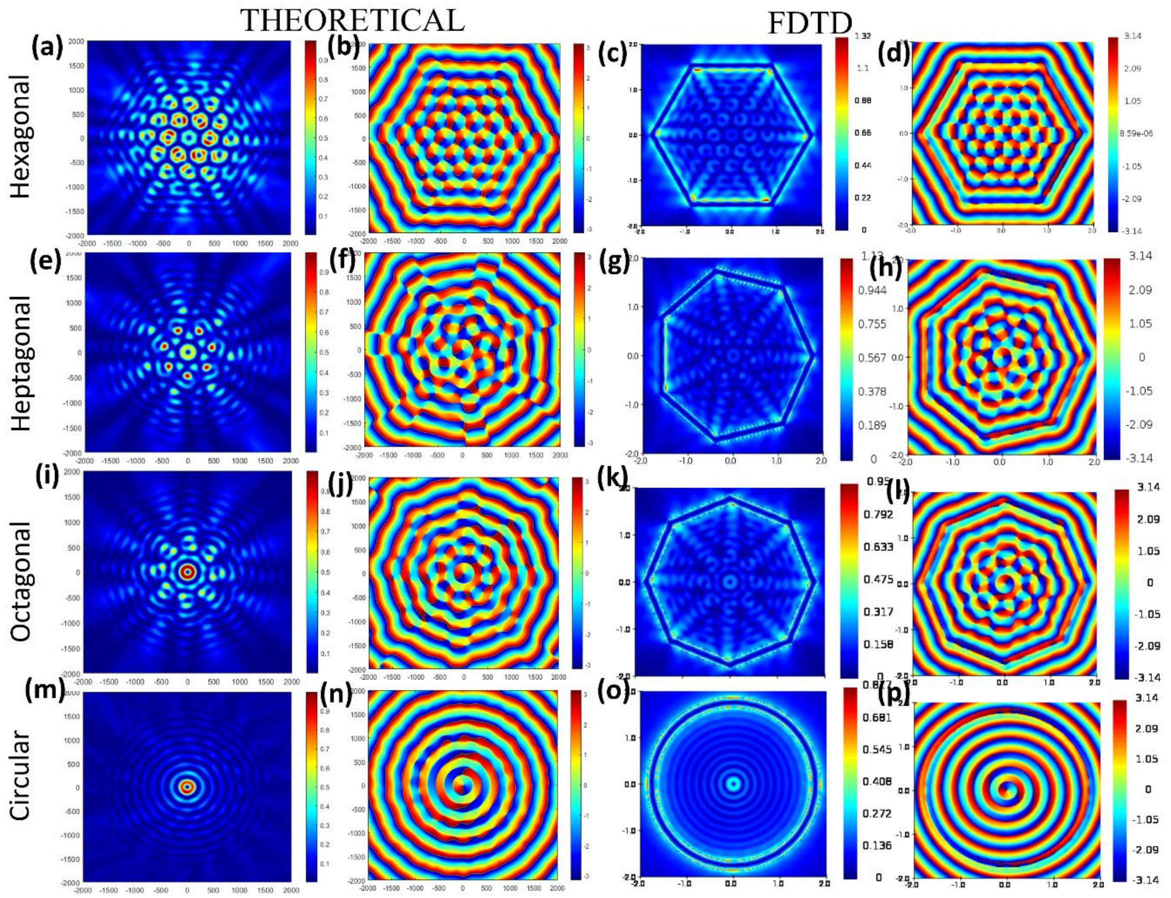

Figure S2. MATLAB plots and FDTD simulations of intensity and phase distribution of different lens structure, i.e., hexagonal lens (a, b, c, d), heptagonal lens (e, f, g, h), octagonal lens (i, j, k, l) and circular lens (m, n, o, p) for LCP polarization at 415 nm wavelength.

S7. Effect of size of lens for linearly polarised illumination in intensity and phase:

In figure S3 and S4, the effect of linearly polarised lights on hexagonal lens has been shown through FDTD simulations and MATLAB plots. No bright spots have been obtained at the centre of the hexagonal lens for both x-polarised and y-polarised illuminations. A “scaling

behaviour" is also obtained in figure S3 and S4 for both x-polarised and y-polarised light.

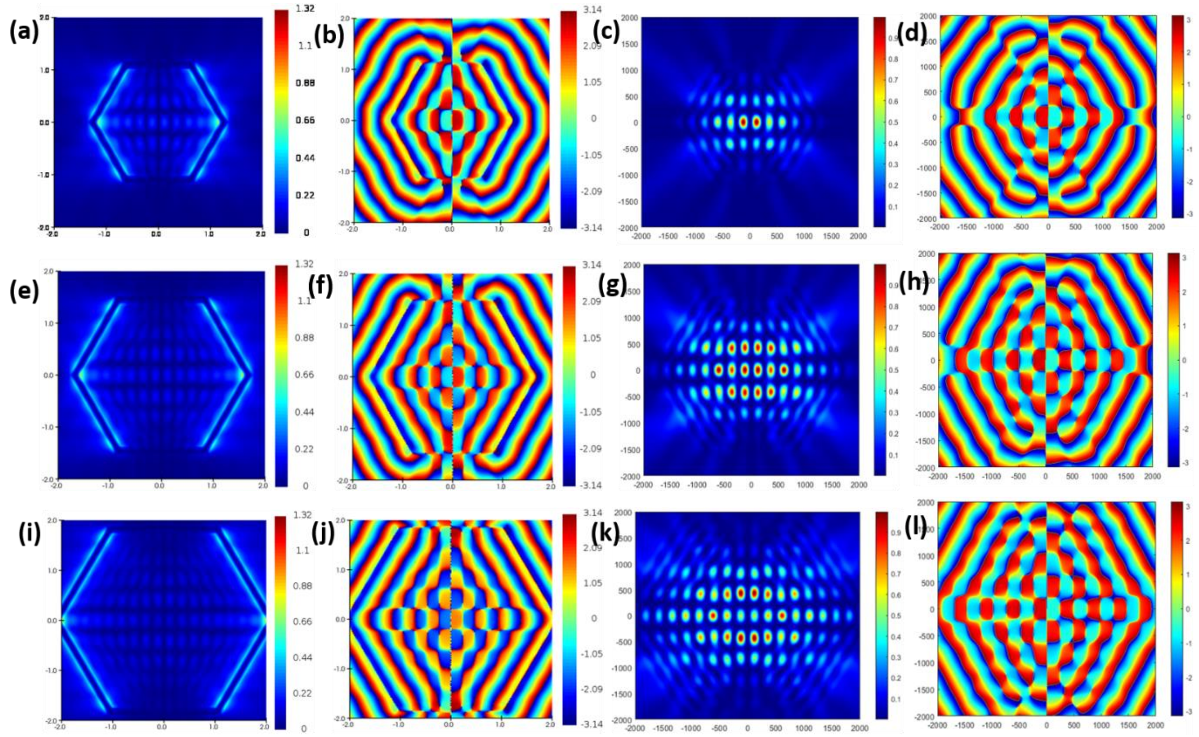

Figure.S3. Intensity and phase distribution of Hexagonal lens in FDTD simulation and in MATLAB for $r_0 = 3\ \lambda_{spp}$ (a, b, c, d), $4\ \lambda_{spp}$ (e, f, g, h) and $5\ \lambda_{spp}$ (i, j, k, l) for x-polarized illumination.

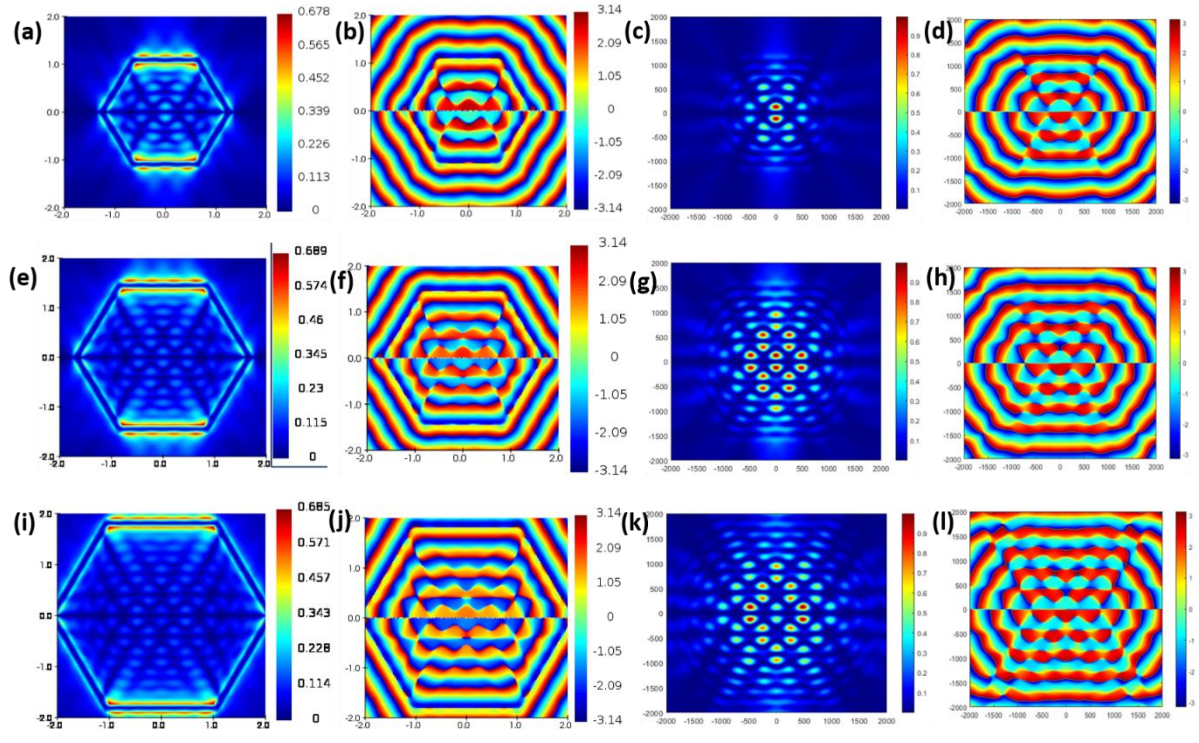

Figure.S4. Intensity and phase distribution of Hexagonal lens in FDTD simulation and in MATLAB for r_0 = 3 λ_{spp} (a, b, c, d), 4 λ_{spp} (e, f, g, h) and 5 λ_{spp} (i, j, k, l) for y-polarized illumination.

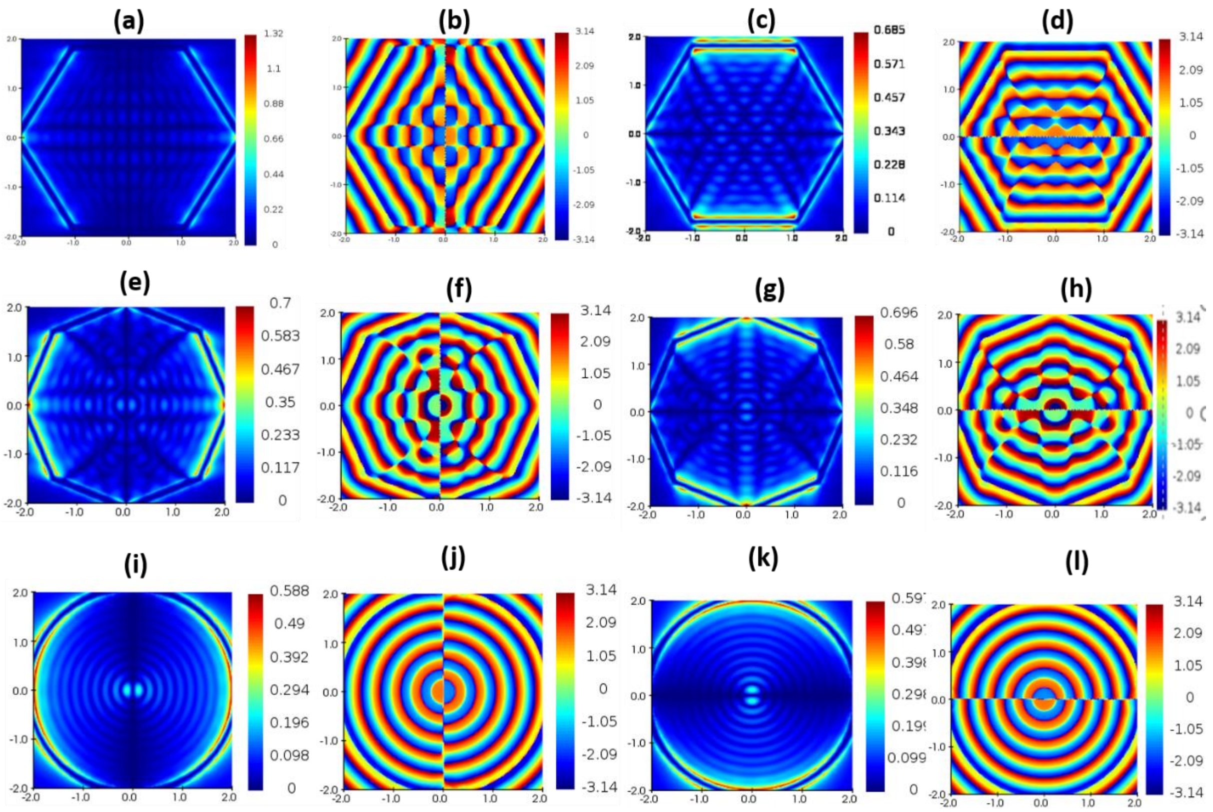

Figure.S5. Intensity and phase distribution of hexagonal lens(a, b, c, d), octagonal lens (e, f, g, h) and circular lens (i, j, k, l) in FDTD simulation for x-polarized(1st 2nd column) and y-polarized (3rd and 4th column) for radius=5 λ_{spp} and illumination wavelength 415 nm in FDTD simulation.

S8. Design features and far-field plots of hexagonal lens:

Far-field studies and design features of best performing plasmonic lens structure has been shown in figure S6. Polarized light is being impinged perpendicularly from the below (the glass side). After generating plasmons in the 2-d surface, the waves get focused at the far-field at focal point at approximately 0.36 µm.[1, 2] The focal point has been determined by far-field super focusing equations.[2] The design parameters of hexagonal lens has been shown schematically in figure S6.

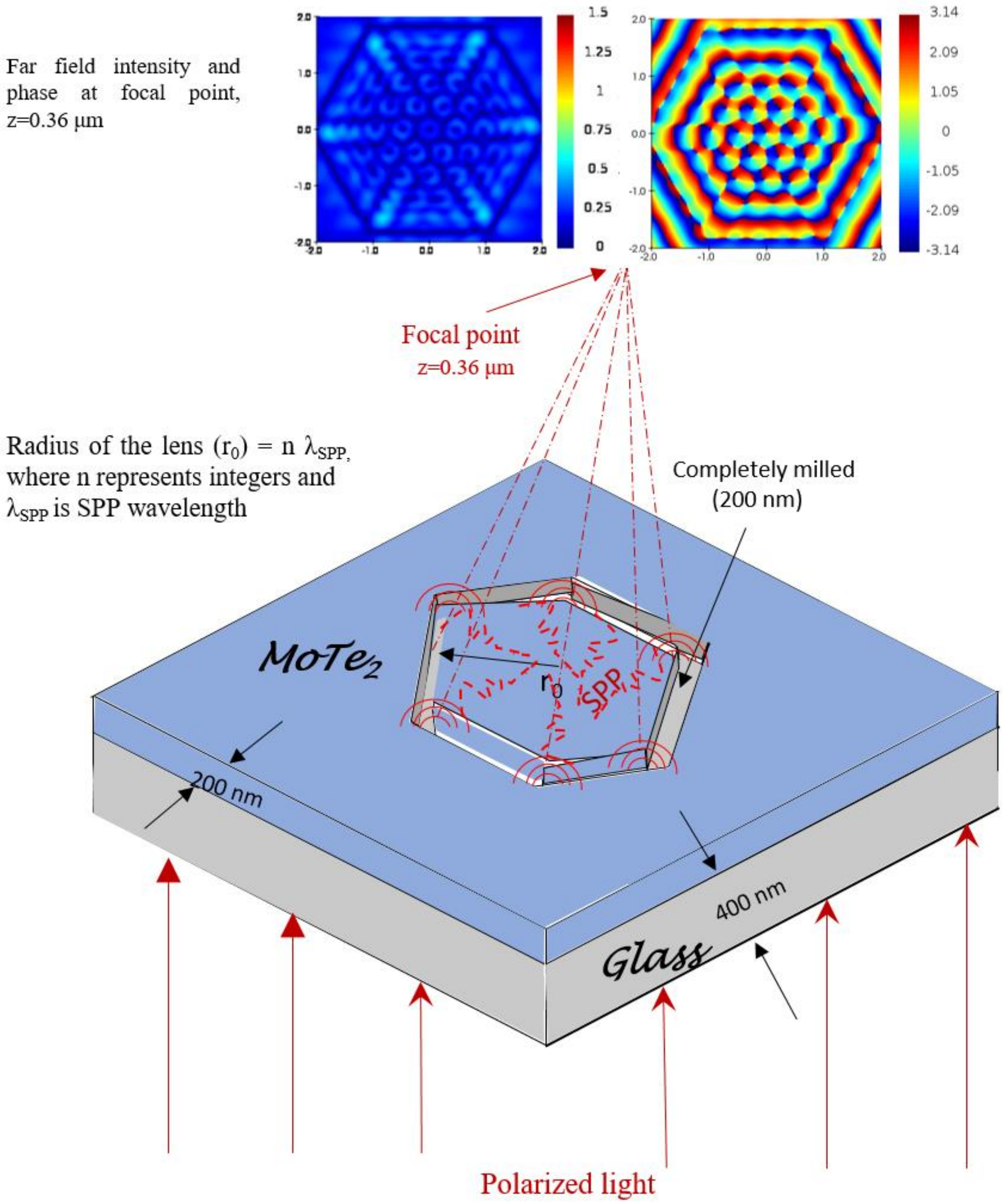

Figure S6: Design features and far field studies of hexagonal plasmonic lens

S9. Investigation of the reasons behind the optical singularities of different plasmonic structures and the algorithm behind the MATLAB plots:

The electrical field equation for polygonal lens structure having number of sides p, has been derived in the manuscript, as:

$$[E_z(\rho,\theta,z)_{\theta' \epsilon [0,2\pi]}]_{Polygonal,RCP}$$

$$= \sum_{n=1}^{p} A_o e^{-k_a z} \int_{\frac{2(n-1)\pi}{p}}^{\frac{2n\pi}{p}} e^{-j\theta'} e^{jk_{spp}\sqrt{\rho^2 + \{r_o \sec\left(\theta' - \frac{(2n-1)\pi}{p}\right)^2\} - 2\rho r_o \sec\left\{\theta' - \frac{(2n-1)\pi}{p}\right) \cos(\theta - \theta')\}}} \, d\theta' \ldots (S9),$$

The equation has been written in a more expanded form.

$$= A_o e^{-k_a z} \left[\left\{ \int_{0}^{\frac{2\pi}{p}} e^{-j\left(\theta' - k_{spp}\sqrt{\rho^2 + \{r_o \sec\left(\theta' - \frac{\pi}{p}\right)^2 - 2\rho r_o \sec\left\{\theta' - \frac{\pi}{p}\right) \cos(\theta - \theta')}\right)} + \right.\right.$$

$$\int_{\frac{2\pi}{p}}^{\frac{4\pi}{p}} e^{-j\left(\theta' - k_{spp}\sqrt{\rho^2 + \{r_o \sec\left(\theta' - \frac{3\pi}{p}\right)^2 - 2\rho r_o \sec\left\{\theta' - \frac{3\pi}{p}\right) \cos(\theta - \theta')}\right)} +$$

$$\int_{\frac{4\pi}{p}}^{\frac{6\pi}{p}} e^{-j\left(\theta' - k_{spp}\sqrt{\rho^2 + \{r_o \sec\left(\theta' - \frac{5\pi}{p}\right)^2 - 2\rho r_o \sec\left\{\theta' - \frac{5\pi}{p}\right) \cos(\theta - \theta')}\right)} +$$

$$\left.\left. \cdots \ldots \ldots + \int_{\frac{2(p-1)\pi}{p}}^{2\pi} e^{-j\left(\theta' - k_{spp}\sqrt{\rho^2 + \{r_o \sec\left(\theta' - \frac{(2p-1)\pi}{p}\right)^2 - 2\rho r_o \sec\left\{\theta' - \frac{(2p-1)\pi}{p}\right) \cos(\theta - \theta')}\right)} \right\} d\theta' \right] \ldots (S10)$$

At any certain position inside the lens structure ρ and θ are constant and r_o, p takes a certain value when the particular lens structure is envisioned. Consequently, it is now obvious that the value of E_z at a particular point (ρ, θ) is obtained after a single integration by solving Equation 9 while holding (ρ, θ) constant. We have to conduct this process relentlessly in as many points as possible in the output monitor range or on to the range we want to investigate to get a clearer image of intensity map. However, we were unable to discover any analytical solutions for the definite integral and had to resort to numerical methods, such as, Simpson's 3/8 rule. The plots agree well with the FDTD simulations.

$e^{-j(\theta' + f(\theta'))}$ is replaced as cos $(\theta' + f(\theta'))$- j sin $(\theta' + f(\theta'))$ in equation S11 and written as below:

$$[E_z(\rho,\theta,z)_{\,\theta'\epsilon[0,2\pi]}]_{Polygonal,RCP} = A_o e^{-k_a z}\left\{\int_0^{\frac{2\pi}{p}} \cos(\theta' + f_1(\theta')) - \mathrm{j}\sin(\theta' + f_1(\theta')) + \int_{\frac{2\pi}{p}}^{\frac{4\pi}{p}} \cos(\theta' + f_2(\theta')) - \mathrm{j}\sin(\theta' + f_2(\theta')) + \int_{\frac{4\pi}{p}}^{\frac{6\pi}{p}} \cos(\theta' + f_3(\theta')) - \mathrm{j}\sin(\theta' + f_3(\theta')) + \cdots \ldots\ldots + \int_{\frac{2(p-1)\pi}{p}}^{2\pi} \cos(\theta' + f_p(\theta')) - \mathrm{j}\sin(\theta' + p(\theta'))\right\} d\theta' \quad (S11)$$

Let's say after integration, the cosine functions produce the values C_1, C_2, C_3…. etc. and the sine functions S_1, S_2 and S_3 for any particular p.

According to the definition, in the co-ordinates of optical singularity points, both the real part and imaginary part of the electric field of the wave are zero turning the phase at those singular points undefined. Mathematically it can be expressed as,

Phase $(Z)_{\text{singularity points}} = tan^{-1}\left[-\frac{S_1+S_2+S_3+\cdots.+S_p}{C_1+C_2+C_3+\cdots+C_p}\right] = undefined.$

For any structure at $(\rho, \theta = 0)$, $S_1 + S_2 + S_3 + \cdots. + S_p = 0$ and $C_1 + C_2 + C_3 + \cdots + C_p = 0$.

For hexagonal lens, at $(\rho, \theta = \pm\lambda_{spp}, \pm\frac{\pi}{3}$ and $0)$ singularity points are obtained for LCP. When the radius of the lens is increased, singularity points are obtained at $\rho = n\lambda_{spp}$. For RCP also, singularities are obtained at only those coordinates only. At those coordinates, electric field at z direction $(E_Z)=0$.

For, circular lens, $E_z = 0$ occurs only at $\rho, \theta = 0{,}0$.

References:

S1. J. Miao, Y. Wang, C. Guo, Y. Tian, J. Zhang, Q. Liu, Z. Zhou and H. Misawa, *Plasmonics*, 2012, **7**, 377-381.

S2. Y. Liu, H. Xu, F. Stief, N. Zhitenev and M. Yu, *Optics express*, 2011, **19**, 20233-20243.
